# Rare case of *Colletotrichum coccodes* causing chronic fungal keratitis in an elderly female: Diagnostic delay and antifungal resistance

**DOI:** 10.4102/sajid.v41i1.809

**Published:** 2026-07-10

**Authors:** Karabo Mathosa

**Affiliations:** 1 Department of Ophthalmology, Groote Schuur Hospital, Cape Town, South Africa

**Keywords:** fungal keratitis, *Colletotrichum coccodes*, antifungal resistance, diabetes mellitus, corneal ulcer, polymerase chain reaction, filamentous fung

## Abstract

Fungal keratitis is a vision-threatening corneal infection, most commonly caused by *Fusarium* and Aspergillus. We report a rare case of keratitis caused by *Colletotrichum coccodes* in an elderly patient with a chronic corneal ulcer refractory to fluconazole. Initial corneal scrapings were negative, delaying diagnosis. Repeat deep biopsy, fungal culture and pan-fungal polymerase chain reaction confirmed *C. coccodes*. The patient was managed with intensive topical amphotericin B and voriconazole, alongside a glycerol-preserved corneal patch graft to provide tectonic support.

**Contribution:**

This case highlights the diagnostic and therapeutic challenges of chronic fungal keratitis and illustrates the limitations of empiric antifungal therapy in the absence of culture-based guidance.

## Introduction

### Background

Fungal keratitis (FK) is a severe ocular infection involving the cornea, caused by filamentous or yeast-forming fungi.^1,2^ Whilst filamentous fungi – most commonly *Fusarium* and *Aspergillus* species – account for the majority of causes, uncommon pathogens such as *Colletotrichum coccodes* can also cause FK, particularly amongst elderly individuals and those with agricultural exposure.^3,4^

*Colletotrichum coccodes* is an exceptionally rare cause of FK, with only a limited number of cases reported in the literature.^5,6^ Delayed pathogen identification and the use of inappropriate antifungal therapy may lead to rapid and severe disease progression, frequently culminating in corneal thinning or perforation.^7^

This report presents a case of chronic FK caused by *C. coccodes* in an elderly woman, emphasising the diagnostic challenges, the influence of diabetes mellitus on disease severity and the clinical utility of molecular diagnostic techniques.

## Case presentation

An 84-year-old woman with no history of ocular trauma or contact lens use presented to our Ophthalmology clinic with a 10-week history of progressively worsening redness, photophobia, epiphora and reduced vision in the left eye. Initial management at an outside Ophthalmology clinic included topical antibiotics and oral fluconazole (400 mg per day); however, no clinical improvement was observed. On further history, the patient reported regular exposure to soil and plant material through home gardening and subsistence farming activities. This environmental exposure represents a potential source of fungal inoculation given the phytopathogenic nature of *Colletotrichum* species.

### Ocular examination

**FIGURE 1:**
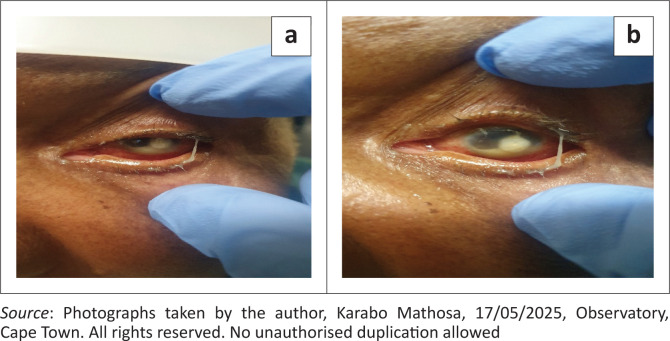
Photographs (a and b) of the left eye of the patient, demonstrating a dense corneal infiltrate with an associated hypopyon.

Figure 1 illustrates the clinical examination findings during examination and were as follows:

- Best-corrected visual acuity: Hand movements in the left eye.
- Cornea: Dense stromal infiltrate with feathery margins, an overlying epithelial defect and a non-mobile hypopyon.
- Intraocular pressure: 10 mmHg, measured using iCare tonometry.
- Posterior segment: Not visualised owing to corneal opacity.

The right eye examination was normal.

### Initial investigations and management

Initial corneal scrapings for bacterial and fungal cultures were negative. Owing to the chronic course and ongoing clinical suspicion for FK, empirical therapy with fortified topical antibiotics was continued, and topical voriconazole was added whilst oral fluconazole was maintained pending culture results.

After 4 weeks without clinical improvement, progressive stromal thinning necessitated emergency placement of a glycerol-preserved corneal patch graft for tectonic support. The excised corneal button obtained during the procedure was submitted for fungal culture and pan-fungal polymerase chain reaction (PCR) analysis.

During this admission, routine laboratory testing revealed previously undiagnosed type 2 diabetes mellitus, evidenced by an elevated Hemoglobin A1c (HbA1c). Metformin therapy was subsequently initiated.

### Laboratory findings and microbiology

Phenotypic identification of cultured fungus: *Colletotrichum coccodes*.

Macroscopic characteristics:

- Colony colour: Grey
- Texture: Fluffy
- Surface (topography): Flat with tuffs and grooves
- Reverse side: Black or dark green
- Growth rate: Moderate
- Optimal temperature: 30 °C
- Pigmentation: None

Microscopic characteristics:

- Hyphae are septate.
- Conidia are oval, some pointed at one end, with septations.
- Conidia are straight, fusiform and attenuated at the ends.

These morphological features are consistent with *Colletotrichum* spp.; however, species-level identification based solely on morphology may be unreliable.^8^

Pan-fungal PCR assay on culture:

- DNA extraction: DNA from culture, yielding a concentration of 77.5 ng/µL.
- PCR amplification: Fungal DNA was amplified using a pan-fungal Internal Transcribed Spacer (ITS) assay.
- Result: *Colletotrichum coccodes* (100% identity; 100% query coverage).

Molecular identification is considered the gold standard for rare filamentous fungi, particularly when culture is slow-growing or inconclusive.^9^ The final report was issued 43 days after specimen collection.

### Management

The treatment regimen was continued based on culture susceptibility:

- Topical amphotericin B four-hourly.
- Topical voriconazole six-hourly.
- Cycloplegics and lubrication for symptomatic relief.

Antifungal susceptibility testing was not performed using formal quantitative methods such as broth microdilution. The organism was identified through culture at the National Health Laboratory Service, with species confirmation by pan-fungal PCR at the National Institute for Communicable Diseases.

Based on known susceptibility patterns of *Colletotrichum* species, the isolate was reported to be susceptible to amphotericin B and voriconazole, and resistant to fluconazole. These findings are consistent with published literature and informed the clinical decision to discontinue fluconazole and continue targeted antifungal therapy.

Fluconazole was discontinued owing to limited efficacy against filamentous fungi.^10^ Treatment continued for 6 weeks, with gradual tapering as symptoms improved. The patient was closely monitored for hepatotoxicity, in anticipation of potential escalation to systemic antifungal therapy should clinical progression occur, and clinical deterioration.

The glycerol-preserved patch graft provided immediate tectonic support in the presence of stromal melt and served as a scaffold for ongoing intensive antifungal therapy. Patch grafts provide effective support in severe FK to preserve globe integrity when penetrating keratoplasty carries a high risk, especially in elderly patients.^11^

### Outcome

Clinical progress during follow-up was as follows:

- At 2 weeks: Minimal reduction in infiltrate size with resolution of hypopyon was observed.
- At 4 weeks: Ongoing stromal melt and residual infiltrate necessitated a glycerol-preserved patch graft. Inflammation subsequently decreased, the graft remained stable, and corneal clarity improved in the surrounding host tissue. Visual acuity improved to counting fingers at 1 m.
- At 6 weeks: Persistent infiltrative activity was noted at the graft–host interface, consistent with ongoing infection rather than a new infectious episode, reflecting the known slow response of filamentous FK, particularly in the context of impaired host immunity.^12^

Antifungal therapy was continued with close clinical monitoring, and gradual stabilisation was achieved thereafter. The graft ultimately remained structurally stable but developed scarring, resulting in a persistently opaque graft.

## Discussion

This case represents a rare instance of chronic FK caused by *C. coccodes*, a plant-associated fungus rarely implicated in human infections. Elderly patients, particularly those with undiagnosed immunosenescence or ocular surface pathology, may be susceptible to atypical pathogens. This case highlights the complex interplay between host factors, diagnostic limitations and pathogen rarity in the management of FK.^6^ The causative organism, *C. coccodes*, is a dematiaceous (darkly pigmented) phytopathogen predominantly associated with plant infections rather than human disease. Its detection in corneal infections is rare and presents important clinical considerations. Clinical features often mimic those of *Fusarium*, including feathery infiltrates and slow progression,^13^ potentially contributing to delayed diagnosis.

### Diabetes mellitus as a risk factor

The patient was newly diagnosed with type 2 diabetes mellitus during her hospital admission, with HbA1c indicating chronic hyperglycaemia. Metformin therapy was initiated. Diabetes is a well-recognised risk factor for infectious keratitis, particularly of fungal origin, owing to impaired corneal wound healing, altered local immunity and increased epithelial fragility. Immunomodulatory defects associated with diabetes create a milieu favourable to invasive fungal pathogens, including rare species. Delayed corneal healing and suboptimal response to conventional antifungal therapy in this case are partly attributable to this systemic comorbidity. Chronic hyperglycaemia is known to exacerbate corneal infections and increase the risk of perforation.^14^

### Limitations of empiric antifungal therapy

The patient was initially treated with oral fluconazole, an antifungal agent primarily effective against yeasts rather than filamentous fungi, such as *Colletotrichum*.^10^ This contributed to a prolonged clinical course without improvement, underscoring the importance of early culture and molecular diagnostics. The initial 4-week fluconazole course potentially exacerbated disease progression via:

- Allowing fungal proliferation.
- Delaying initiation of appropriate antifungal therapy.
- Promoting stromal melt.
- Increasing the need for surgical intervention.

Voriconazole and amphotericin B, administered in combination in this case, provide broad-spectrum coverage against most filamentous fungi, including *Colletotrichum* species.^15^ Combination therapy with amphotericin B and voriconazole has been reported to improve outcomes in filamentous FK, particularly in rare dematiaceous organisms. Published case reports of *Colletotrichum* keratitis similarly describe prolonged treatment courses and the frequent need for surgical intervention to achieve infection control. The clinical response observed in our patient is consistent with these findings.

In South Africa, the epidemiology of FK differs from that reported in temperate regions, with filamentous fungi accounting for the majority of cases. These organisms, including *Fusarium, Aspergillus* and rare dematiaceous species such as *Colletotrichum*, demonstrate a propensity for early stromal and anterior chamber invasion.

Whilst initial empiric therapy in this case included oral fluconazole, this agent is largely ineffective against filamentous fungi. In high-risk presentations – particularly in elderly patients, those with environmental exposure, or in regions with a known predominance of filamentous pathogens – early consideration of systemic antifungal therapy such as oral voriconazole may be justified.

Systemic voriconazole has demonstrated the ability to achieve therapeutic concentrations in the aqueous humour, potentially addressing deeper fungal penetration that topical agents alone may not adequately reach.

Earlier initiation of systemic therapy may therefore reduce disease progression and the need for surgical intervention in selected cases.

### Role of molecular diagnostics and repeated sampling

Initial cultures and scrapings were non-diagnostic, a common occurrence in FK.^16^ This may reflect low fungal load, prior antimicrobial therapy or sampling error. Repeat sampling, particularly deep corneal biopsies, enhances diagnostic yield.^17^ Definitive diagnosis was achieved through PCR analysis, confirming *C. coccodes*. This reinforces the value of molecular diagnosis in culture-negative, treatment-refractory keratitis, particularly in elderly patients or those with immunocompromised status.^9^

### Surgical intervention: Glycerol-preserved patch graft

**FIGURE 2:**
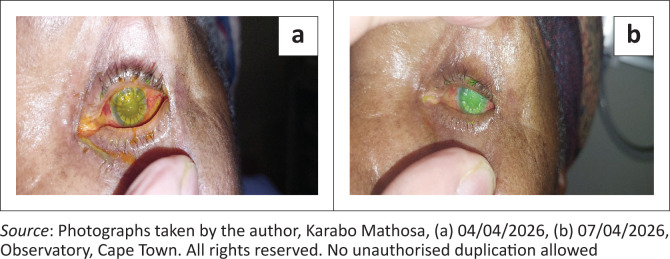
Photographs (a and b) of an eye post application of a glycerol-preserved corneal patch graft.

Progressive corneal thinning with impending perforation necessitated application of a glycerol-preserved corneal patch graft to reinforce globe integrity. Glycerol-preserved tissue offers several advantages in infectious keratitis, including immediate availability, reduced antigenicity and resistance to secondary infection.^11^ The graft, as shown in Figure 2, provide tectonic support during antifungal therapy, permitting infection resolution and subsequent epithelialisation. Whilst therapeutic penetrating keratoplasty remains the gold standard in some cases of severe FK, patch grafting is a valuable interim strategy in elderly patients or those unsuitable for major surgery.

In this case, the glycerol-preserved patch graft served a dual role: providing tectonic support in the setting of stromal melt and facilitating therapeutic debulking of infected tissue. Additionally, submission of the excised corneal button enabled definitive microbiological diagnosis.

### Emerging pathogenicity of *Colletotrichum* species

Whilst traditionally considered a plant pathogen, *Colletotrichum* is increasingly detected in ocular infections, particularly in tropical and subtropical regions.^3^ The mechanisms underlying ocular pathogenicity remain unclear but likely involve trauma-related inoculation (including microtrauma), immune compromise and environmental exposure. Fewer than 20 cases of *Colletotrichum* keratitis have been reported globally, with *Colletotrichum dematium* and *Colletotrichum gloeosporioides* being the most frequently identified species.^3,5^ This report represents one of the very few documented cases of keratitis caused by *C. coccodes* in an elderly patient.

### Clinical implications and learning points

This case underscores several important considerations:

- Chronic or non-healing corneal ulcers in elderly or diabetic patients should prompt early consideration of FK – even in the absence of trauma or conventional risk factors.
- Fluconazole is ineffective against filamentous fungi and should not be administered empirically.
- Negative culture results do not exclude fungal infection, making repeat sampling essential.
- Molecular diagnostics, including PCR and ITS sequencing, substantially improve the identification of rare fungal pathogens.
- Glycerol-preserved patch grafts provide effective tectonic support in severe FK.
- Early diagnosis is often missed owing to a non-specific clinical presentation and the low susceptibility of routine fungal cultures. In this case, the specimen was collected on 18 March 2025, and the final report was issued on 30 April 2025, representing a delay of 43 days.

## Conclusion

This case underscores the importance of considering rare fungal pathogens, including *C. coccodes*, in chronic, non-healing keratitis. Delayed diagnosis and inappropriate empiric antifungal therapy may result in poor clinical outcomes. Early recognition, advanced diagnostic methods and targeted therapy are critical for infection resolution and vision preservation.
